# School-Based Health Education Targeting Intestinal Worms—Further Support for Integrated Control

**DOI:** 10.1371/journal.pntd.0002621

**Published:** 2014-03-13

**Authors:** Franziska A. Bieri, Yue-Sheng Li, Li-Ping Yuan, Yong-Kang He, Darren J. Gray, Gail M. Williams, Donald P. McManus

**Affiliations:** 1 QIMR Berghofer Medical Research Institute, Herston, Brisbane, Queensland, Australia; 2 The School of Population Health, The University of Queensland, Brisbane, Queensland, Australia; 3 Hunan Institute of Parasitic Diseases, WHO Collaborating Centre for Research and Control on Schistosomiasis in Lake Region, Yueyang, People's Republic of China; Natural History Museum, United Kingdom

In an editorial published in PLOS Neglected Tropical Diseases in August 2009, Peter Hotez [1] wrote:

“*Given the extremely modest costs, the rapid health impact, and the resulting high rates of economic return, we cannot afford further delays in implementing large-scale NTD [neglected tropical disease] control measures.*”

Our report, which describes success in preventing soil-transmitted helminth (STH) infections in Chinese schoolchildren through the use of a health education package that includes a 12-minute cartoon, “The Magic Glasses,” may thus prove timely. The results from the cluster randomized intervention trial, conducted in 38 rural Chinese schools and involving 1,718 children, showed that the video-based health education package had 50% efficacy in preventing new STH infections after treatment [2]. This study established proof of principle that health education can indeed increase knowledge and change behavior, resulting in fewer intestinal worm infections. However, we have a ways to go before we can show broad application, and we appreciate that these findings will require further validation in other epidemiological and cultural settings.

With a third of the world's population infected [3], STHs are the most widespread and disabling chronic infections globally and the most common of the neglected tropical diseases (NTDs). The most recent estimate (2010) of the worldwide burden of infection with STHs is 5.2 million disability-adjusted life years (DALYs) [4]. Children have the greatest risk of morbidity due to STHs [5], which have a particularly debilitating effect on their health and cognitive development, causing anemia, malnutrition, stunted growth, and learning delays [3]. Almost half of the global disease burden due to these worm infections is borne by children 5 to 14 years of age [5]. A recent study has shown that this age group can contribute up to 50% of the *Ascaris lumbricoides* burden in the community [6].

In 2012, the announcement of the World Health Organization (WHO) roadmap [7] galvanized public and private support in the fight against the NTDs. Major pharmaceutical companies; the Bill & Melinda Gates Foundation; the governments of the United States, United Kingdom, and United Arab Emirates; and the World Bank have announced substantial funding to accelerate the control, elimination, and possibly the eradication of the NTDs. Measures mainly include sustaining or expanding current mass drug administration (MDA) programs to meet the United Nations (UN) Millennium Development Goals by 2020 and promoting research and development of new drugs and drug targets by sharing expertise and compounds. All partners endorsed the “London Declaration on Neglected Tropical Diseases,” through which they pledged new levels of collaborative effort and tracking of progress in tackling ten of the 17 NTDs currently on WHO's list [8]. The WHO strategy for STH control is to treat all preschool- and school-age children and women of childbearing age living in endemic areas. Treatment frequency depends on the STH prevalence in a particular community. When the prevalence of intestinal worm infection exceeds 20%, mass treatment should be given to major risk groups once a year; two treatments per year are recommended when the prevalence is greater than 50%. In addition to MDA, WHO recommends health education and the provision of clean water and adequate sanitation to reduce reinfection [9].

Significant progress has been made in controlling STH infections over the past 10 years, with over 300 million preschool-age and school-age children dewormed in 2011 (representing 30% of all children in the world at risk of STH infection due to living in endemic areas) [10]. However, the UN Millennium Development Goal of treating 75% of school-age children at risk of STH infection by 2010 was not achieved. In the WHO roadmap to overcome the global impact of NTDs, released in January 2012, the goal for STH was reset to 75% treatment coverage for children in endemic areas by 2020 [7]. Even if this ambitious goal is achieved by 2020, concerns regarding the effectiveness and sustainability of MDA remain. The efficacy of the available drugs, albendazole and mebendazole, is high for *A. lumbricoides*, moderate to high for hookworm, but low for *Trichuris trichiura* [11]–[13]. For the last 20 years, rapid reinfection rates after treatment cessation have been reported repeatedly for intestinal helminths [14]. For example, in a high-prevalence setting in Pemba Island, Tanzania, in 1995, pretreatment infection levels were reached only 6 months after treatment was stopped [15]. In another, more recent study from Sri Lanka, even after 10 years of mass chemotherapy, the STH prevalence rebounded to pretreatment levels after MDA was terminated [16].

There is also growing concern about the potential development of parasite resistance to albendazole and other anthelminthics as a result of continued treatment pressure [13]. It is considered inevitable that drug resistance will develop in human nematodes, given the many examples of drug-resistant nematode species infecting sheep and other livestock due to extensive drug use [17]–[19]. Furthermore, community enthusiasm for MDA often wanes, especially when the community is not engaged in the program [20]. This can result in poor compliance and donor fatigue in areas where treatment needs to be repeated regularly [21]–[25]. In addition, maintaining a supply of anthelminthic drugs in remote areas can be difficult, and this may distract from other priorities in resource-poor areas, weakening the local health care system [26].

The benefit of MDA has been intensely debated after the recent publication of a second edition of a Cochrane review by Taylor-Robinson and colleagues in 2012, which summarized the effects of deworming on nutritional indicators, hemoglobin, and school performance in 42 randomized controlled trials (RCTs) comprising a total of 65,000 participants [27]. The review concluded that there is insufficient reliable information to determine whether deworming has an effect on nutritional indicators, hemoglobin, and school performance and, hence, the justification for programs consisting of only deworming is questionable [27]. Whether this perceived limited benefit of MDA is due to lack of sustainability, poor compliance, variable efficacy of the deworming drugs used in mass treatment programs, or a combination thereof was not discussed in the review, and these issues require addressing in the future. The report has stimulated intense debate within the NTD community, although its methods of analysis and study selection criteria have been criticized by MDA advocates. This debate is ongoing and being continually updated in a *PLOS Medicine* blog that can be visited at http://blogs.plos.org/speakingofmedicine/2012/07/18/should-deworming-policies-in-the-developing-world-be-reconsidered.

Taking all this information into account, interventions preventing STH reinfection, such as improvements in hygiene through health education, are urgently required to augment the effect of chemotherapy as part of an integrated multicomponent approach to control. Chemotherapy temporarily reduces morbidity and infection prevalence, whereas preventive interventions, such as health education or improved sanitation, prevent reinfection, thereby reducing incidence. In the long term, a combination of the two approaches will lead to a reduction in the number of treatment cycles required for effective control, thereby reducing the treatment pressure and resulting in a more sustainable approach to control [20], [28].

China is in an ideal position to undertake an integrated public health strategy that combines MDA with health education and improved water, sanitation, and hygiene (WASH) for the control of STHs. It has already successfully pioneered multicomponent integrated control strategies, which include health education, for other NTDs such as lymphatic filariasis [29] and schistosomiasis [30], [31]. Due to the intense economic development, improved sanitation, and raised living standards, STH prevalence has decreased considerably in eastern China, whereas endemic hot spots remain in remote rural areas of Yunnan, Sichuan, and Guizhou provinces [32]. Worldwide, China's economic and political influence is increasing rapidly, and expectations have already been raised for China to take the lead in transferring its NTD control and elimination know-how and to provide support for NTD control in sub-Saharan Africa [33].

We concur with others [34], [35] that the only way to achieve sustainable control of STH infections and, more generally, to achieve the UN Millennium Development Goals is through integrated control efforts in a multicomponent strategy, including chemotherapy, improved WASH, and health education, that addresses the social and ecological causes of STH, and not stand-alone treatment-based control of a number of different NTDs. Treatment-based control, or targeting multiple diseases with one MDA program, is also often referred to as integrated control [36], [37] but is not to be confused with the multicomponent integrated control strategy as described above and by Utzinger and colleagues [35]. The need for health education and additional public health measures, including novel, effective, and easy-to-use educational control tools, has repeatedly been advanced [35], [37]–[44]. The literature indicates that strategies involving health education inducing behavior change have been poorly implemented in practice [45], and evidence-based control strategies that address the social, cultural, and behavioral dimensions of disease are urgently required [45]. Furthermore, their cost effectiveness and the cost per DALY averted need to be determined. The effective video-based educational package we have developed and tested [2] complements the current strategy for the control of STHs as advocated by the WHO. With culturally sensitive adaptation and appropriate resourcing, this tool has the potential to be incorporated into current ongoing deworming programs, not only in China [46] and other parts of Southeast Asia but also in sub-Saharan Africa, with implications for the future control of STHs globally.
